# Genome-wide identification of genes probably relevant to the adaptation of schizothoracins (Teleostei: Cypriniformes) to the uplift of the Qinghai-Tibet Plateau

**DOI:** 10.1186/s12864-017-3703-9

**Published:** 2017-04-20

**Authors:** Wei Chi, Xufa Ma, Jiangong Niu, Ming Zou

**Affiliations:** 1 College of Fisheries, Huazhong Agricultural University, Wuhan, China; 2 Key Laboratory of Freshwater Animal Breeding, Ministry of Agriculture, Wuhan, China; 3 Fisheries Research Institute of Xinjiang Uygur Autonomous Region, Urumqi, China

**Keywords:** Schizothoracins, Polyploid, High-altitude adaptation, Positive selection

## Abstract

**Background:**

Molecular adaptation to the severe environments present during the uplift of the Qinghai-Tibet Plateau has attracted the attention of researchers. The divergence of the three specialization groups of schizothoracins (Primitive, Specialized and Highly Specialized) may correspond to the three phases of plateau uplift. Based on the transcripts of representative species of the three specialized groups and an outgroup, genes in schizothoracins that may have played important roles during the adaptation to new environments were investigated.

**Results:**

The contigs of *Gymnodiptychus dybowskii* and *Schizothorax pseudaksaiensis* were compared with those of *Gymnocypris przewalskii ganzihonensis* and the outgroup *Sinocyclocheilus angustiporus*, and 5,894 ortholog groups with an alignment length longer than 90 nt after deleting gaps were retained. Evolutionary analyses indicated that the average evolutionary rate of the branch leading to the Specialized group was faster than that of the branch leading to the Highly Specialized group. Moreover, the numbers of gene categories in which more than half of the genes evolved faster than the average values of the genome were 117 and 15 along the branches leading to the Specialized and Highly Specialized groups, respectively. A total of 40, 36, and 55 genes were likely subject to positive selection along the branches leading to the Primitive, Specialized and Highly Specialized groups, respectively, and many of these genes are likely relevant to adaptation to the cold temperatures, low oxygen concentrations, and strong ultraviolet radiation that result from elevation.

**Conclusions:**

By selecting representative species of the three groups of schizothoracins and applying next-generation sequencing technology, several candidate genes corresponding to adaptation to the three phases of plateau uplift were identified. Some of the genes identified in this report that were likely subject to positive selection are good candidates for subsequent evolutionary and functional analyses of adaptation to high altitude.

**Electronic supplementary material:**

The online version of this article (doi:10.1186/s12864-017-3703-9) contains supplementary material, which is available to authorized users.

## Background

Understanding the molecular mechanisms of adaptation is one of the central goals of evolutionary biology. In particular, molecular events underlying adaptation to extreme environments, such as high altitude, have attracted widespread attention. Along with the uplift of the land, native species must cope with harsh conditions, such as cold temperatures, low oxygen concentrations, and strong ultraviolet radiation [1, 2]. Many new tribes have emerged due to this adaptation, and they are valuable models for understanding the molecular mechanisms of high-altitude adaptation.

The genetic basis of high-altitude adaptations in humans has been the most studied. Genomic studies targeting Tibetan populations have shown that the genes *EGLN1* and *PPARA*, which are significantly associated with the decreased hemoglobin phenotype, were positively selected in the highland population [3]. Further investigation revealed that genes associated with human reproductive disorders and the biological process categories “response to DNA damage stimulus” and “DNA repair” showed a distinct allele frequency pattern of copy number variable region (CNVR) distribution in Tibetans [4]. In Andeans, *EGLN1* also exhibited evidence of positive selection [5]. In Ethiopian highlanders, genomic analysis revealed that a number of candidate loci were associated with hemoglobin levels relating to high-altitude adaptation [6].

The deer mouse (*Peromyscus maniculatus*), which is native to the Andean highlands, is the most studied high-altitude species other than humans. Deer mice have a relatively low hemoglobin content [7], but variations in the globin genes seem to be the basis for the increased oxygen affinity of the hemoglobin and faster transport of oxygen [8]. Moreover, deer mice use fats as a high percentage of their metabolic fuel in order to retain carbohydrates for small bursts of energy [9]. The yak (*Bos grunniens*) is the most important domesticated animal of Tibetan highlanders, and it thrives only at high altitudes. Comparison of the genome sequences of yaks with those of cattle indicated an expansion of gene families related to sensory perception and energy metabolism in yaks and an enrichment of protein domains involved in sensing the extracellular environment and hypoxic stress [10]. Researchers also found that genes related to hypoxia and nutrition metabolism showed positive selection [10]. The ground tit (*Pseudopodoces humilis*) is endemic to the northern Himalayas, and genome sequencing revealed positive selection of genes related to cardiac function and the expansion of the corresponding gene families in this species [11].

However, molecular events underlying high-altitude adaptations of organisms living in water, such as fish, are rarely reported. Water can weaken the intensity of ultraviolet radiation, and the oxygen availability in water differs from that in air; therefore, we assumed that the adapted fish genes may be somewhat different from those of land animals. Yang et al. compared orthologous genes among a schizothoracine fish, *Gymnodiptychus pachycheilus*, and several model fish with available genome sequences and suggested that many of the genes that are associated with energy metabolism and hypoxia were subjected to positive selection during its plateau adaptation [12]. This finding was supported by a functional verification study [13]. However, given that schizothoracins are polyploid and other model fishes are diploid [14], together with the fact that the divergence times among these fishes are large, comparing orthologs among schizothoracins and closely related polyploid cyprinids should be more effective to gain insights into the molecular mechanisms of plateau adaptation in schizothoracins.

**Fig. 1:**
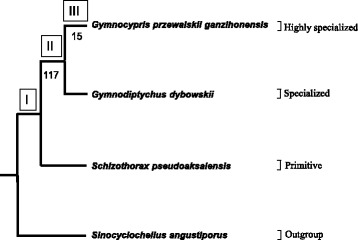
Phylogenetic relationships of the species used in this report. *G. dybowskii* and *S. pseudoaksaiensis* were collected from the Ili River, which is located in Sinkiang, northwestern China; *G. p. ganzihonensis* and the outgroup species *S. angustiporus* were respectively collected from the Ganzi River in Qinghai province and from the Huangnihe River in Yunnan province, previously [32, 33]. I, II and III represent branches leading to the Primitive, Specialized and Highly Specialized groups, respectively. Numbers below branches denote the number of fast-evolving gene categories with the same GO term or KEGG pathway assigned along branches II and III, respectively

The Qinghai-Tibet Plateau is sometimes called “the Third Pole” and “the Roof of the World” because it is the highest and largest plateau in the world. The uplift of the Plateau can be divided into phases, although there are many debates regarding this process [15, 16]. Schizothoracins are endemic to the Qinghai-Tibet Plateau, and they are assumed to originate from the original barbins. By comparing morphological traits, such as scales, pharyngeal teeth and barbels, Cao et al. divided schizothoracins into three specialized groups: Primitive, Specialized and Highly Specialized (Fig. 1) [17]. They concluded that the variation among these traits corresponded to the continuous uplift of the plateau, which has been supported by molecular phylogenetic analysis [18].

To understand the molecular events underlying the adaptations of schizothoracins to different phases of plateau uplift, representative fish from the three specialized groups of schizothoracins and an outgroup were selected (Table 1); by comparing orthologs among these species, genes that were potentially relevant to the adaptation to the uplift of the Qinghai-Tibet Plateau during the emergence of Primitive, Specialized and Highly Specialized fish (branches I, II and III, respectively, in Fig. 1) were identified. Our analyses may provide a deeper understanding of the process of high-altitude adaptation of schizothoracins.

**Table 1:** Summaries of the short reads of the transcriptome sequencing projects for the polyploid cyprinids used in this report

*Species*	*Specialized groups*	*Origin*	*SRA accession number*	*Read length*	*Platform*	Total nt
*G. p. ganzihonensis*	Highly Specialized	SRA	SRR1542351	100	HiSeq2000	7.1G
			SRR1542357	100	HiSeq2000	7.2G
*G. dybowskii*	Specialized	This report	SRR3496460	250	MiSeq	1.4G
			SRR3501163	125	HiSeq2000	9.4G
*S. pseudaksaiensis*	Primitive	This report	SRR3496459	250	MiSeq	1.5G
			SRR3496380	125	HiSeq2000	9.5G
*S. angustiporus*	Outgroup	SRA	SRR788094	75	GAII	1.1G
			SRR788096	95	HiSeq2000	9.3G

## Methods

### Data acquisition

Transcripts of *G. dybowskii* and *S. pseudoaksaiensis* were generated in this report. Using gill nets, four live samples of each species were collected from the Ili River, which is located in Sinkiang, northwestern China (Additional file 1: Figure S1). For each individual, total RNA from the brains and livers was extracted using TRIzol reagent (Transgene Company, Illkirch-Graffenstaden, France) according to the manufacturer’s protocol. All of the specimens were euthanized with 300 mg/L of tricaine methanesulfonate (MS 222) before tissue collection. RNA degradation and contamination were preliminarily monitored using 1% agarose gels. A NanoPhotometer spectrophotometer (IMPLEN, CA, USA) was then used to confirm the purity of the RNA, and a Qubit RNA Assay Kit and a Qubit 2.0 Flurometer (Life Technologies, CA, USA) were employed to measure the concentration of RNA. The RNA integrity number (RIN) of each sample was assessed using the RNA Nano 6000 Assay Kit of the Agilent Bioanalyzer 2100 system (Agilent Technologies, CA, USA), and these were all greater than 8.0. For each species, total RNA from the brain and liver was mixed and used for subsequent library construction with a TruSeq Stranded mRNA Library Prep Kit as described elsewhere, except that the preferentially selected cDNA fragments were 500 bp in length [19]. Sequencing was performed using an Illumina HiSeq 2000 platform, and 125-bp paired-end reads were generated. Given that these two fish are polyploid and that longer short reads may result in more accurate assembly, the same libraries were also sequenced using an Illumina MiSeq platform, and 250-bp paired-end reads for each species were obtained. For the purpose of evolutionary analyses, short reads of the transcriptome sequencing of another polyploid schizothoracins, *G. p. ganzihonensis*, and *S. angustiporus* as the outgroup, were downloaded from the NCBI SRA (http://www.ncbi.nlm.nih.gov/sra, Table 1 and Additional file 2: Table S1).

### Quality control, sequence assembly and annotation

For each species, each run of short reads was subject to quality control using the NGS QC Toolkit v2.3.3 [20] with the default settings. This toolkit can perform a quality check and filter high-quality next-generation sequencing data automatically. Next, all of the processed data were combined and assembled using Trinity r20131110 [21] with the default settings for each species. For the *de novo* assembly of the transcriptome for each species, TransDecoder v2.0 (sourceforge.net/projects/transdecoder/) was used to predict the probable open reading frames. For *G. dybowskii*, contigs were compared to the proteins of *Danio rerio* (zebrafish) deposited in the KEGG database with BLASTX 2.3.0+ [22], and corresponding KEGG pathways and gene ontology (GO) terms were retrieved using the online tool KOBAS2.0 with the default settings [23]. For simplicity, all of the subsequent functional analyses were based on the annotations for *G. dybowskii*.

### Ortholog assignments and sequence alignments

Based on the predicted protein sequences, orthologous groups among *S. angustiporus, S. pseudaksaiensis*, *G. dybowskii* and *G. p. ganzihonensis* were identified using Inparanoid 4.1 [24] and Multiparanoid [25] with default settings. Inparanoid can detect in-paralogs with a confidence value, the results were fed into Multiparanoid, and the in-paralogs with the best confidence values were selected as orthologous. The nucleotide sequences of each orthologous group were aligned to the predicted amino acid sequence of *G. dybowskii* using GeneWise 2-4-1 [26], followed by a series of customized Perl scripts to extract the matched coding regions and to generate the proper alignment format for subsequent analyses. Then, SWAMP 31-03-14 was used to mask the unreasonably high rates of nonsynonymous substitutions, which were likely caused by sequencing, assembly, or alignment errors [27]. Finally, alignments longer than 90 nt after deleting the gaps were retained.

### Gene categories with accelerated evolutionary rates

Analyses of gene categories with accelerated evolutionary rates along each lineage were performed following the protocol of Yang et al. [28]. For each ortholog group, the branch-specific values of Ka, Ks and Ka/Ks were estimated using codeml, which is included in the PAML4 software package [29]. The genome-wide average values of Ka, Ks and Ka/Ks along each branch were estimated using 10,000 concatenated alignments that were constructed from 150 randomly chosen ortholog groups. Next, the GO categories with more than 20 assigned orthologous groups were selected, and pairwise comparisons of the Ka/Ks values with the average values for the branches leading to Specialized and Highly Specialized groups (branches II and III shown in Fig. 1) were implemented using a binomial test. For corrections of multiple comparisons, the method of Benjamini and Hochberg was performed to reduce false positives [30].

### Detecting candidate genes subject to positive selection

Positive selection on a few codons along branches I, II and III was detected using the optimized branch-site model [31]. By setting each of the three branches as the foreground branch, the likelihood ratio values for the model, which allows sites to be under positive selection on the foreground branch, and the null model, in which sites may evolve neutrally and under purifying selection, were calculated. Based on the likelihood ratio values and a Chi-square distribution, the significance of the differences between the two nested models for all ortholog groups was obtained and adjusted using the method of Benjamini and Hochberg for multiple tests [30]. To confirm the convergence of the Markov process, the aforementioned tests were performed twice with different starting values.

## Results and discussion

### Sequencing, assembly and alignment of orthologs

For *G. dybowskii*, the Illumina MiSeq and HiSeq2000 sequencing platforms respectively generated 11,550,142 and 75,226,074 paired-end reads (Table 1 and Additional file 2: Table S1). For *S. pseudaksaiensis*, the corresponding procedures generated 11,769,716 and 76,383,862 reads. After quality control, more than 74 M paired-end reads totaling more than 10 G nt for both of the species were retained and subjected to subsequent *de novo* assembly. Based on the combined data generated from different sequencing platforms, a total of 139,382 and 114,997 contigs with N50 lengths of 2407 nt and 2283 nt were obtained for *G. dybowskii* and *S. pseudaksaiensis*, respectively. In previous projects sequencing the transcriptomes of polyploid cyprinids, the amounts of data generated for the two species were larger than that for *G. pachycheilus* [12] but were smaller than those for *S. angustiporus* and *G. przewalskii* [32, 33]. However, we hypothesized that the longer length of short reads, especially of those generated by the MiSeq platforms, may lead to some compensation.

For *G. p. ganzihonensis*, short reads from the gills and kidneys were combined, producing a total of 210,085 contigs with an N50 length of 1447 nt. As expected, when these short reads with different origins were assembled separately, a much larger number of contigs with a shorter N50 length was generated [32]. For *S. angustiporus*, short reads from the whole eye and brain were combined and assembled, which generated 100,762 contigs with an N50 length of 621 nt. However, based on the same data, the number of contigs generated by Meng et al. was 156,118, with an N50 length of 534 nt [33]. The different numbers of contigs and different N50 lengths may be due to our use of different quality control tools and different versions of *de novo* assembly software.

A total of 5894 orthologous groups with an alignment length longer than 90 nt after removing gaps was obtained and subject to subsequent evolutionary analyses. Most of the alignment lengths were shorter than 400 nt, and the average and median lengths of the alignments were 219 and 192 nt, respectively (Additional file 3: Figure S2). The relatively short alignment lengths may have resulted from the incomplete sequencing of transcripts. We expect that a much greater number of alignments could be obtained if more tissues were sampled and longer reads were generated, given that all fish species that were subjected to this analysis were polyploid.

### Fast-evolving gene categories

The average Ka/Ks value of the branch leading to the Highly Specialized group was significantly lower than that of the branch leading to the Specialized group (*p*-value < 2.2e-16, Binomial test). By contrast, the average values of Ka and Ks were significantly larger in branches leading to the Specialized group (*p*-value < 2.2e-16, Binomial test). The same pattern was found when comparing the Ka, Ks and Ka/Ks values between terminal branches leading to *G. dybowskii* and *G. p. ganzihonensis* (*p*-value < 2.2e-16, Binomial test). The somewhat unexpected larger Ks and smaller Ka/Ks values for *G. p. ganzihonensis* may be due to the following three reasons: the intensive ultraviolet radiation may accelerate the mutation rates of the entire genome; divergence of morphological traits may lag behind sequence divergences; and shorter read lengths generated by Illumina HiSeq sequencing platforms may result in more paralog assemblies.

Several gene categories with the same GO term or KEGG pathway assigned were identified as fast-evolving gene categories along branches II and III, as shown in Fig. 1. There were 117 and 15 fast-evolving gene categories for branches II and III, respectively (fdr < 0.05, Binomial test, Additional file 4: Table S2). Unexpectedly, the evolutionary rates of the GO categories, which were probably relevant to adaptation to high altitudes, such as “response to hypoxia”, “ATP metabolic process” and “response to UV” [28], were not significantly accelerated in our analyses. This phenomenon likely results from at least three causes: in the waters of the Tibetan Plateau, the ultraviolet intensity may be lower than that of air, and the water may contain higher levels of oxygen; the evolutionary rates of these genes probably accelerated during the adaptation to the first phase of plateau uplift; and many of these genes may also be expressed in other organs, such as the skin, or may have been lost when performing ortholog assignments. Moreover, we considered that more fast-evolving gene categories were represented in branch II and may be the result of relaxed selection, given that branch III is believed to undergo stronger selective pressure. Further investigation of recruiting transcripts from various tissues of more representative species of the three main groups of schizothoracins and outgroups should be used to test these hypotheses.

### Candidate genes probably subject to positive selection

**Fig. 2:**
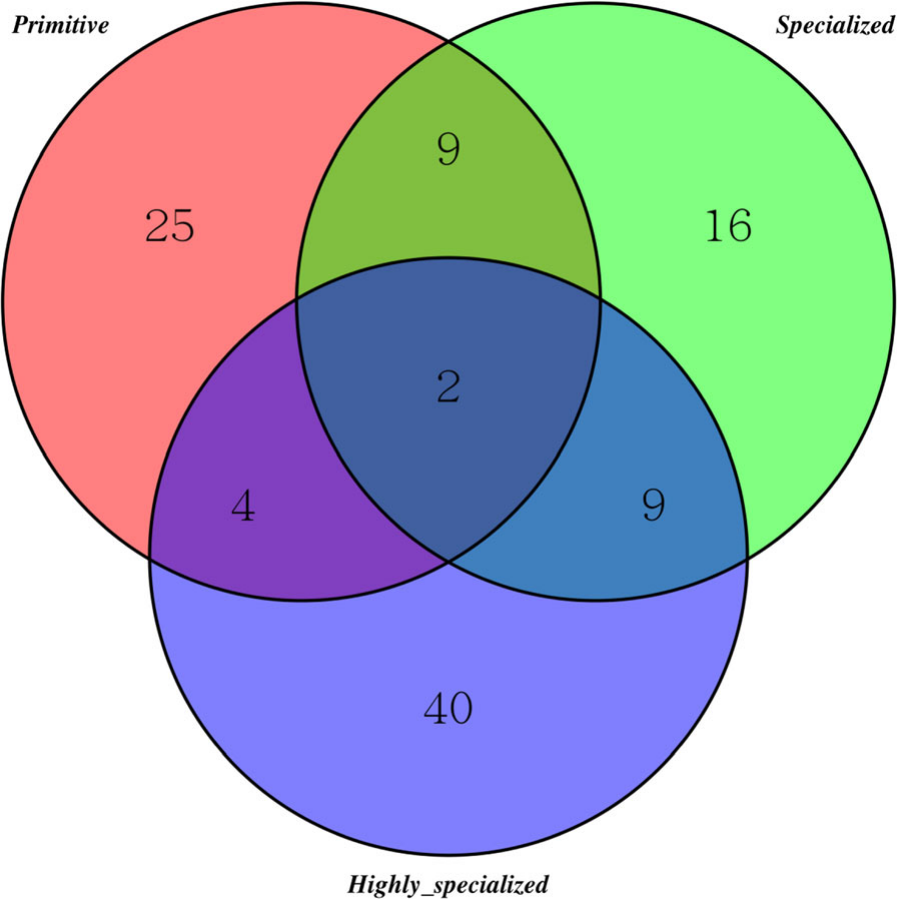
Venn diagram of the overlap between the candidate genes subject to positive selection along the branches leading to the Primitive, Specialized and Highly Specialized groups of Schizothoracins (branchesI, II and III in Fig. 1)

Likelihood ratio tests indicated that 40, 36, and 55 genes were probably subject to positive selection along branches I, II, and III, respectively (fdr < 0.05, Additional file 5: Table S3). Interestingly, several genes were subject to continuous positive selection during plateau uplift (Fig. 2). Specifically, two of these genes were subject to positive selection along the branches leading to all of the Primitive, Specialized, and Highly Specialized groups (hereafter termed PSH for convenience), and the numbers of genes subject to positive selection along the branches leading to the Primitive and Specialized (PS), Specialized and Highly Specialized (SH), and Primitive and Highly Specialized (PH) groups were 9, 9, and 4, respectively. The numbers of lineage-specific genes subject to positive selection along the branches leading to the Primitive (P), Specialized (S) and Highly Specialized (H) groups were 25, 16, and 40, respectively.

One of the two PSH genes was highly similar to “ecotropic viral integration site 5b” (*evi5b*) in zebrafish, which harbors a coiled-coil region that resembles that of structural maintenance of chromosomes (SMC) proteins. These proteins are core components of the condensing and cohesin complexes responsible for regulating chromosomal condensation, pairing and segregation in eukaryotes [34]. Given that the fish that were subjected to analyses in this report were all polyploid, we assumed that *evi5b* probably played an important role in their diploidization. Another gene, annotated as “supervillin b,” seems to be involved in cytokinesis during cell division [35]. However, why it was subject to positive selection throughout the course of plateau-adaptation in schizothoracins is presently unknown.

Two of the PS genes, “protein jagunal homolog 1-A” (*jagn1*) and “polypyrimidine tract-binding protein 2” (*ptbp2*), were subject to positive selection, which is not unexpected, given that the former is an immune gene that can regulate neutrophil function in microbial pathogenesis [36] and the latter is a reproductive protein that may function specifically in the male germline [37]; these two kinds of genes usually undergo more adaptive evolution than other genes [38, 39]. Notably, two PS genes, “mycophenolic acid acyl-glucuronide” and “solute carrier organic anion transporter family member 3a1” (*slco3a1*), which are related to the transport of organic anions [40, 41], were also subject to positive selection, but the reasons for that selection remain unknown.

The light intensity increases with the elevation of the plateau, and one of the SH genes, “guanine nucleotide-binding protein beta-1” (*gnb1*), which is involved in the pathway “Phototransduction”, was subject to positive selection, likely resulting from adaptation to the greater light intensity. Moreover, intense ultraviolet light can cause DNA damage and even worse, skin cancer [42]. Thus, the “dab2 interacting protein” (*dab2ip*) gene, which suppresses the development of many cancer types [43], likely appeared among the SH genes for this reason. Meanwhile, some genes relevant to energy metabolism, for example, the “2-oxoglutarate dehydrogenase” (*ogdh*) [44] and *atp6v0a1* genes, participate in the “oxidative phosphorylation” pathway and probably contributed to adaptation to the reduced water temperature.

P genes may have been important for adaptation to the first phase of plateau uplift. These genes include several relevant to adaptation to hypoxia, low temperature, and high-intensity ultraviolet light. First, hypoxia can reduce the activity of “arylsulfatase b” (*arsb*) [45], but it is a tumor suppressor and is presumably associated with carcinogenesis at low levels [46–48]; therefore, *arsb* has evolved to adapt to the low-oxygen environment. The formation and maintenance of the cristae structure of the inner mitochondrial membrane is largely based on the mitochondrial contact site and cristae-organizing system (MICOS), and “MICOS complex subunit MIC19” (*mic19*) may have adapted to maintain the normal mitochondrial structure and function during hypoxia [49]. *Mic19*, together with “cytochrome c-type heme lyase” (*cchl*), which is involved in electron transfer processes [50], and “*creg1*”, which promotes cardiomyogenesis [51], may be indirectly relevant to hypoxia. Second, two other P genes, *bag1* and a member of the immunoglobulin superfamily that is homologous to several cell adhesion molecules, “cell surface glycoprotein MUC18” (*mcam*), may have evolved to adapt to high-intensity ultraviolet light, which can cause DNA damage and induce cancer. The reason for this adaptation may be that the products of *bag1* can form complexes with the anti-apoptotic protein bcl-2 and can thereby contribute to rendering cells more resistant to apoptosis, and altered *bag1* expression levels can be detected in some cancerous cells [52], whereas *mcam* cannot be detected in normal melanocytes but can be detected in primary melanomas and is highly expressed in metastatic melanoma cells [53]. Moreover, “bromodomain-containing 2” (*brd2*) plays a critical role in adipogenesis [54] and may be adapted to low temperatures.

The third phase of uplift may have been the fastest, and the height has increased by 3000 m since approximately 2.6 Myr ago [55]. During this process, a number of genes were supposed to be under positive selection. The “endoplasmic reticulum transmembrane prolyl 4-hydroxylase” (*p4h-tm*) gene can hydroxylate the subunit of hypoxia-inducible factor (hif) [56]. In normoxia, hydroxylation of 2 critical proline residues can rapidly degrade hif because the hif-subunit isoforms hif-1 and hif-2 are synthesized constitutively. However, in hypoxia, hydroxylation is inhibited, allowing hif to escape degradation and regulate hypoxia-related genes [57]. Moreover, *p4h-tm* can regulate erythropoietin production, hepcidin expression, and erythropoiesis [57]. Therefore, we suggest that the modification of *p4h-tm* may contribute to the adaptation to hypoxic waters in the Qinghai-Tibet Plateau. The protein “*nibrin*” plays an important role in the initial recognition of DNA damage during the recruitment of proteins responsible for DNA double-strand breaks repair [58]. “Tumor protein d54” (*tpd52l2*) is a member of the D52-like family, and these proteins have been predicted to interact with each other to regulate cell proliferation [59]. “Tumor protein D52” (*tpd52*) is frequently overexpressed in several carcinomas and may be the co-expressed *tpd52l2* [60]. The two aforementioned cancer-related genes were positively selected, perhaps as the result of adaptation to the increasingly high-intensity ultraviolet light associated with the uplift of the plateau. Notably, the H genes included several genes related to the physiological function of the nervous system, such as “neurexin-1β”, which binds with the postsynaptic membrane protein neuroligin-1 and plays a central role in the formation of synapses in the central nervous system [61]; “kinesin-like protein kif1a” (*kif1a*), a unique monomeric motor for the anterograde axonal transport of synaptic vesicle precursors [62]; “wnt-7a”, which preferentially stimulates excitatory synapse formation and function [63]; “rest corepressor 2” (*rcor1*), which is co-expressed with zmynd8 and interacts to form a complex that might be involved in the regulation of neural differentiation in the nervous system [64]; and “alsin”, which is particularly abundant in motor neurons and is involved in the development of axons and dendrites [65]. Potassium channels are found in most cell types and control a wide variety of cell functions, including modulating neuronal signaling in the brain and the peripheral nervous system, as well as regulating cell volume and the flow of salts across epithelia [66]. “Voltage-gated potassium channel subunit beta-1” (*kcnab1*) may markedly influence the gating mode of potassium channels [67], along with “ammonium transporter rh type b” (*rhbg*), which may be a major ammonium transporter in vertebrates [68], were also included as H genes. The modification of these genes relevant to ion transport and physiological function of the nervous system may have resulted from adaptation to the changed environment.

## Conclusions

Using the assembled transcripts, which were based on short reads generated in this report and others downloaded from a public database, this study identified several genes that likely corresponded to the adaptation of three specialized groups of schizothoracins to the three phases of plateau uplift. The short reads generated for *G. dybowskii* and *S. pseudaksaiensis*, especially the longer short reads generated using the Illumina MiSeq sequencing platform, should be valuable resources for subsequent analyses of polyploid cyprinids. However, to more thoroughly understand the molecular events of adaptation, additional representative species should be evaluated, and further comprehensive sequencing of the transcripts should be performed.

## Additional files

Additional file 1: Figure S1. Sampling sites of the studied species. The triangle represents the sampling site of *G. dybowskii* and *S. pseudaksaiensis* collected in this study. The circle and diamond represent the sampling sites of *G. p. ganzihonensis* and the outgroup species *S. angustiporus* collected previously [32, 33]. The map was adopted from Sogou Map (map.sogou.com). (PPT 137 kb)

Additional file 2: Table S1. Specifics of the short reads of the transcriptome sequencing projects for the polyploid cyprinids used in this report. (XLS 16 kb)

Additional file 3: Figure S2. Distribution of alignment lengths after deleting gaps. The solid line indicates the average length, and the dotted line indicates the median length. (PPT 34 kb)

Additional file 4: Table S2. Gene categories showing the accelerated evolutionary rates along the branches leading to the Specialized and Highly Specialized groups of schizothoracins (branches II and III in Fig. 1). (XLS 40 kb)

Additional file 5: Table S3. Profiles of candidate genes subject to positive selection along branches leading to the Primitive, Specialized and Highly Specialized groups of schizothoracins (branchesI, II and III in Fig. 1). (XLS 39 kb)

## Abbreviations

GO: Gene ontology
H: Highly Specialized group of schizothoracins
KEGG: Kyoto encyclopedia of genes and genomes
P: Primitive group of schizothoracins
S: Specialized group of schizothoracins
SRA: Sequence read archive of NCBI
