# Supplementary figures and images for: Genome-wide identification of genes probably relevant to the adaptation of schizothoracins (Teleostei: Cypriniformes) to the uplift of the Qinghai-Tibet Plateau
Source: BMC Genomics. 2017 Apr 20;18:310. doi: 10.1186/s12864-017-3703-9 (PMC5397779; doi:10.1186/s12864-017-3703-9)

## Slide 1
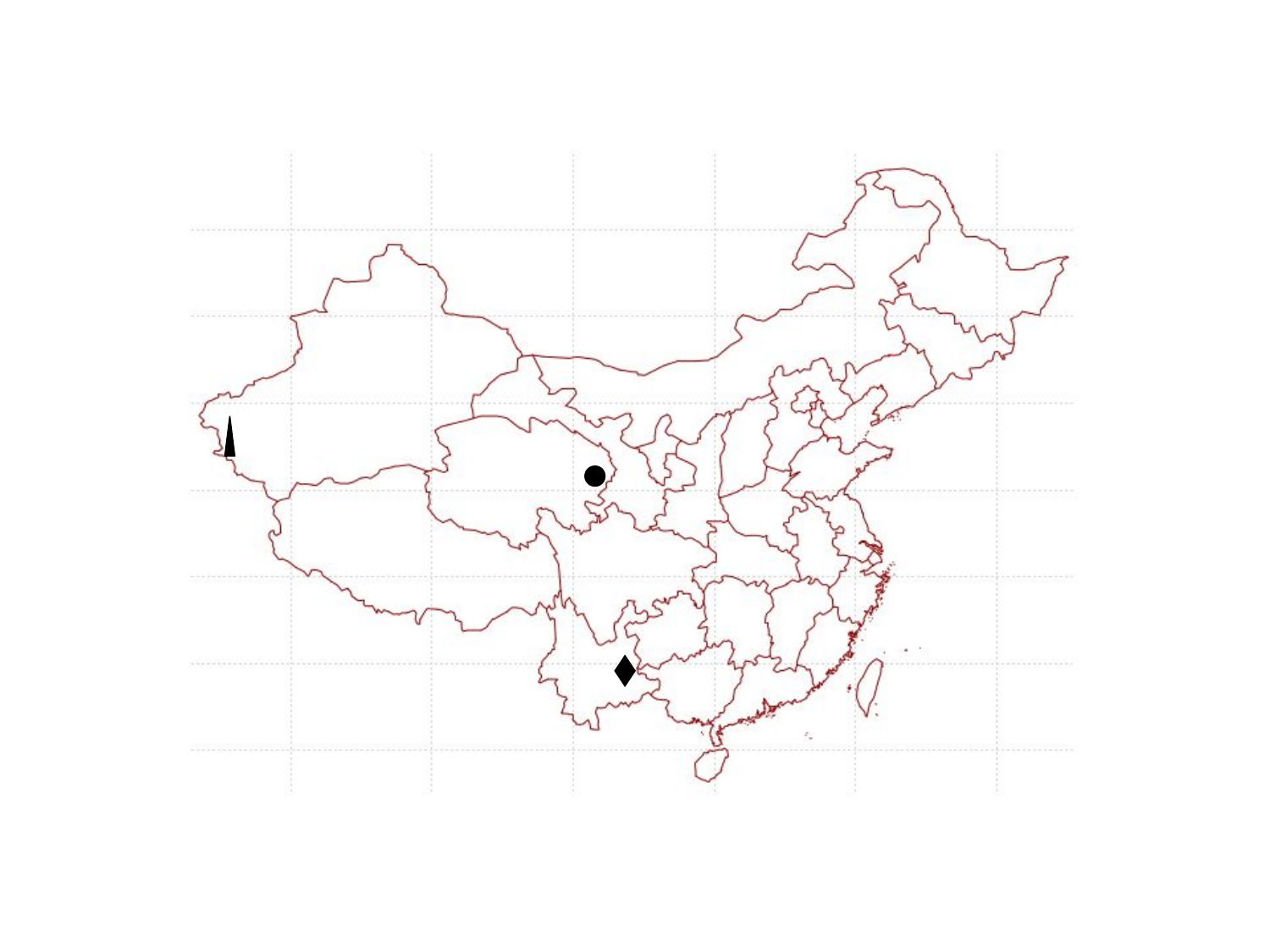

#

Supplement: Supplementary file 1 — Sampling sites of the studied species. The triangle represents the sampling site of G. dybowskii and S. pseudaksaiensis collected in this study. The circle and diamond represent the sampling sites of G. p. ganzihonensis and the outgroup species S. angustiporus collected previously [32, 33]. The map was adopted from Sogou Map (map.sogou.com). (PPT 137 kb) [file 12864_2017_3703_MOESM1_ESM.ppt]

## Slide 1
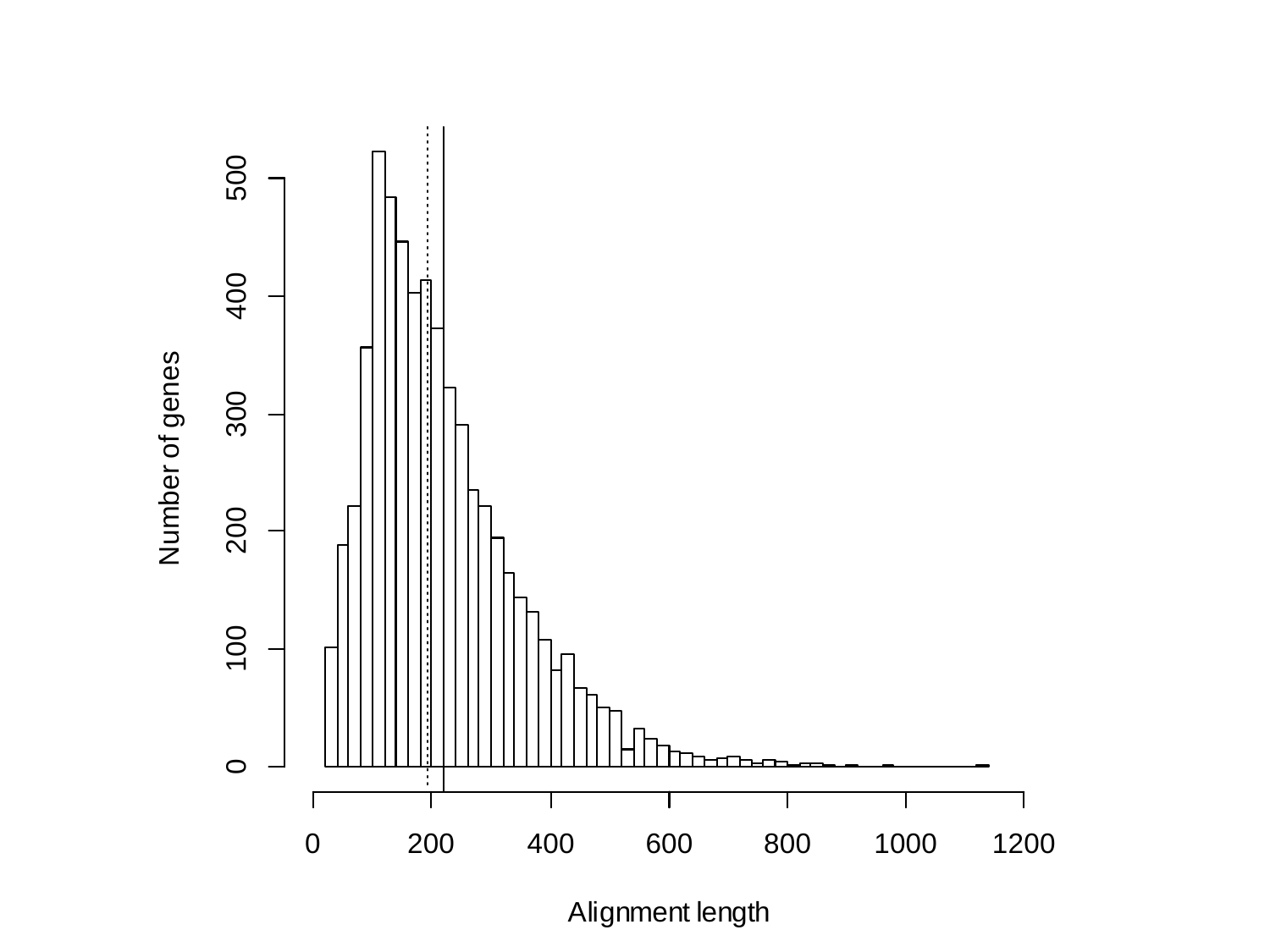

Supplement: Supplementary file 3 — Distribution of alignment lengths after deleting gaps. The solid line indicates the average length, and the dotted line indicates the median length. (PPT 34 kb) [file 12864_2017_3703_MOESM3_ESM.ppt]
